# Cross-trait genetic enrichment between GERD and psychiatric disorders in East Asian populations

**DOI:** 10.3389/fgene.2026.1770067

**Published:** 2026-02-03

**Authors:** Zhihao Gao, Xianjin Wang, Zekun Liu, Fen Hu, Yidi Zhou, Kalim Ullah, Ruiwei Wang, Meng Zhang, Xiao Chang, Yongsen Wang

**Affiliations:** 1 First Clinical Medical College, Shandong University of Traditional Chinese Medicine, Jinan, Shandong, China; 2 College of Medical Information and Artificial Intelligence, Shandong First Medical University, Jinan, Shandong, China; 3 School of Pharmacy, Yantai University, Yantai, Shandong, China; 4 Department of Anesthesiology, Shandong Provincial Hospital Affiliated to Shandong First Medical University, Jinan, Shandong, China; 5 Gastroenterology Department, Affiliated Hospital, Shandong University of Traditional Chinese Medicine, Jinan, Shandong, China

**Keywords:** conditional false discovery rate, cross-trait enrichment, gastroesophageal reflux disease, genetic correlation, psychiatric disorders

## Abstract

Gastroesophageal reflux disease (GERD) exhibits significant epidemiological comorbidity with psychiatric disorders, yet their shared genetic architecture remains poorly characterized in East Asian populations. Leveraging ancestry-specific genome-wide association study (GWAS) summary statistics from East Asian cohorts, we employed linkage disequilibrium score regression and conditional false discovery rate (condFDR) approaches to investigate cross-trait genetic enrichment between GERD and major psychiatric disorders, including major depressive disorder (MDD), schizophrenia (SCZ), and bipolar disorder (BIP). We identified significant genetic correlations between GERD and both MDD (r_g_ = 0.49, *P* = 0.03) and SCZ (r_g_ = 0.25, *P* = 0.02), but not with BIP. Through condFDR analysis, two novel loci were discovered: rs3980178 near *MEIS1*(associated with GERD-MDD pleiotropy) and rs9844126 near *ZBTB20*(associated with GERD-SCZ pleiotropy). These loci are implicated in neurodevelopment, autonomic regulation, and neural circuit formation, providing mechanistic insights into the gut-brain axis. Our findings demonstrate that cross-trait genetic enrichment significantly enhances locus discovery for GERD in underpowered East Asian GWAS and reveal ancestry-specific genetic links between gastrointestinal and psychiatric phenotypes.

## 1 Introduction

Gastroesophageal reflux disease (GERD) is a highly prevalent condition with substantial clinical and public health impact. Although historically viewed as a disorder driven by esophageal motility dysfunction and mucosal injury, accumulating evidence points to a more complex etiology involving epithelial integrity, immune activation, sensory processing, and central neural regulation (Azer and Goosenberg, 2025). In parallel, GERD shows strong epidemiological comorbidity with several psychiatric disorders, including major depression, anxiety disorders, post-traumatic stress disorder, and attention-deficit/hyperactivity disorder (Ding et al., 2025). These co-occurrence patterns suggest that shared biological pathways may contribute to susceptibility across both gastrointestinal and mental health domains.

Genome-wide association studies (GWAS) have identified numerous loci associated with GERD and psychiatric traits; however, most existing studies have been conducted in European-ancestry populations (An et al., 2019; Anurag et al., 2024). The genetic architecture of complex diseases often differs across ancestral groups due to variation in allele frequencies, linkage disequilibrium (LD) structure, and environmental exposures. As a result, the transferability of European-derived findings to East Asian populations remains limited, and ancestry-specific genetic determinants of GERD and psychiatric disorders are still poorly understood. Given the unique LD patterns and allele distributions in East Asians, leveraging population-specific GWAS offers an opportunity to uncover novel shared mechanisms that may not be detectable in European datasets.

Traditional single-trait GWAS may lack the statistical power to fully resolve the polygenic overlap between GERD and psychiatric disorders. Recently developed conditional false discovery rate (condFDR) approaches provide a powerful framework for enhancing genetic discovery by exploiting cross-trait enrichment patterns (Smeland et al., 2019). By conditioning GERD association statistics on the strength of association with a genetically correlated trait, condFDR increases sensitivity to detect true GERD risk loci, including those with sub-genome-wide effects. This strategy is particularly advantageous in East Asian populations, where sample sizes for individual traits are still modest compared to large European consortia, yet shared polygenic structure can be leveraged to boost effective statistical power (Wang et al., 2025; Zhi et al., 2025; Song et al., 2025; Yang L. et al., 2025).

In this study, we performed a comprehensive condFDR analysis of GERD and multiple psychiatric disorders specifically within East Asian populations. Our primary aim was to identify novel GERD susceptibility loci through cross-trait genetic architecture. By integrating East Asian GWAS resources with cross-trait enrichment models, our work seeks to expand the catalog of GERD-associated loci, illuminate ancestry-specific biology, and clarify the genetic links between gastrointestinal and psychiatric phenotypes.

## 2 Results

### 2.1 Meta-analysis of GERD in East Asian populations using Taiwan precision medicine initiative and biobank Japan datasets

We first analyzed two available GERD GWAS datasets generated from East Asian cohorts the Taiwan Precision Medicine Initiative (TPMI; 47,138 cases and 271,934 controls (Yang HC. et al., 2025)) and the BioBank Japanese (BBJ; 948 cases and 177,516 controls (Sakaue et al., 2021)). Across both datasets, no variants reached genome-wide significance (*P* < 5 × 10^−8^), consistent with the modest sample sizes and lower power relative to large European-ancestry GERD GWAS. To increase statistical power, we performed a fixed-effect inverse-variance–weighted meta-analysis combining the TPMI and BBJ datasets. The combined analysis similarly did not identify any genome-wide significant loci, and the Manhattan plots showed no signals approaching the conventional significance threshold. These findings highlight the challenge of locus discovery for GERD in East Asian populations using single-trait GWAS alone, and indicate that alternative analytic strategies leveraging cross-trait genetic information may be required to improve detection of true susceptibility loci.

### 2.2 Genetic correlation between GERD and psychiatric disorders in East Asians

We next evaluated whether GERD shares heritable genetic architecture with major psychiatric disorders in East Asian populations. Using cross-trait linkage disequilibrium score regression (LDSC), we estimated genome-wide genetic correlations between our East Asian GERD meta-analysis (TPMI + BBJ) and three psychiatric GWAS datasets for which East Asian–specific summary statistics were available. For major depressive disorder (MDD), we extracted the East Asian subset from the recent multi-ancestry meta-analysis (Meng et al., 2024). Schizophrenia (SCZ) associations were obtained from a dedicated East Asian GWAS comprising 22,778 cases and 35,362 controls (Lam et al., 2019). For bipolar disorder (BIP), we used the East Asian subset of the largest available GWAS to date, which includes 158,036 cases and ∼2.8 million controls across ancestries (O’Connell et al., 2025).

LDSC results showed that GERD exhibited a significant genetic correlation with MDD (r_g_ = 0.4866, SE = 0.2249, *P* = 0.0305) and with SCZ (r_g_ = 0.2457, SE = 0.1044, *P* = 0.0185), indicating the presence of shared polygenic components between GERD and these psychiatric disorders (Table 1). By contrast, the genetic correlation between GERD and BIP was not significant (r_g_ = 0.1902, SE = 0.1639, *P* = 0.2458). Given the limited number of psychiatric traits examined, these genetic correlation analyses were performed to characterize patterns of genome-wide sharing rather than to conduct formal hypothesis testing across a large trait space. Notably, only MDD and SCZ showed nominally significant correlations with GERD, whereas BIP did not, suggesting trait-specific rather than pervasive genetic overlap. These results were therefore used to motivate subsequent conditional analyses rather than interpreted as standalone locus-level evidence. These findings also suggest that, within East Asian populations, GERD liability shares modest but statistically meaningful heritable overlap with depressive and schizophrenia-related risk, whereas bipolar disorder does not exhibit detectable genome-wide sharing with GERD.

**TABLE 1:** Genetic correlation between GERD and psychiatric traits.

Trait1	Title2	r_g_	SE	*P*
GERD	Bipolar disorder	0.1902	0.1639	0.2458
	Major depressive disorder	0.4866	0.2249	0.0305
	Schizophrenia	0.2457	0.1044	0.0185

Trait1/Trait2: Traits analyzed. rg: genetic correlation. SE: standard error. *P*: Significance *P*-value.

### 2.3 Cross-trait conditional FDR analysis identifies shared risk loci between GERD and psychiatric disorders in East Asians

**FIGURE 1:**
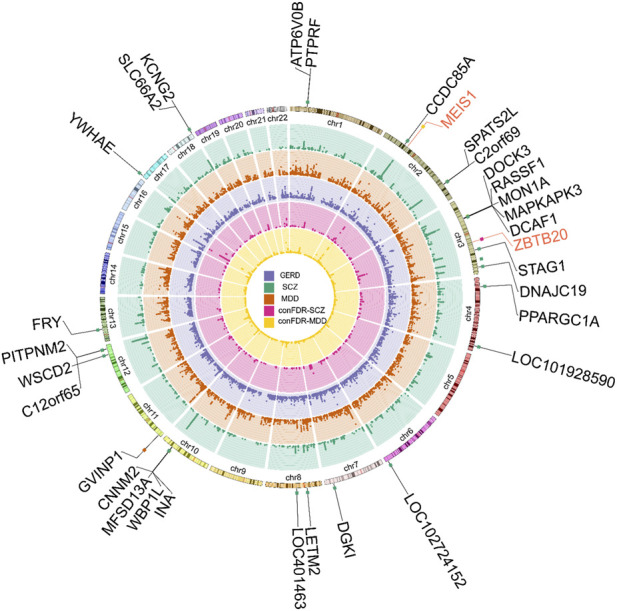
Overview of genome-wide signals across GERD and psychiatric disorders. This circos plot presents genome-wide association patterns for GERD and two psychiatric disorders (MDD and SCZ) in East Asians, along with cross-trait associations identified using the condFDR framework. From the outermost to innermost rings, the plot displays genomic coordinates, −log_10_(*P*) values for each trait, and condFDR tracks that highlight regions of cross-phenotype association. Genes shown in black correspond to loci previously reported to be associated with GERD, whereas genes in red represent loci newly identified through the condFDR analysis.

Given the significant genome-wide genetic correlations between GERD and both MDD and SCZ, we next applied the condFDR framework to determine whether cross-trait enrichment could augment locus discovery in East Asian populations. Despite the modest power of available GERD GWAS datasets, conditioning on genetically correlated psychiatric traits uncovered two loci that reached significance (Figure 1; Table 2).

**TABLE 2:** condFDR analysis between GERD and psychiatric traits.

Trait 1	Trait 2	SNP	CHR	BP	A1	A2	*P_cfdr*	*P1*	BETA1	*P2*	BETA2	Gene.refGene	cytoBand
GERD	Major depressive disorder	rs3980178	2	67023335	A	C	1.17E-05	1.49E-06	0.0394	0.005873	0.0426	*MEIS1*	2p14
	Schizophrenia	rs9844126	3	114852509	G	T	0.0463956128355677	1.52E-05	0.036	0.02398	−0.0398017	*ZBTB20*	3q13.31

Trait 1/Trait 2: Traits analyzed. SNP: Lead single-nucleotide polymorphism (SNP) representing each genome-wide significant locus. CHR: Chromosome on which the SNP, is located. BP: Base-pair position (hg19/hg38 as specified). A1/A2: Effect allele (A1) and non-effect allele (A2). *P*_*cfdr*: The significance level *P*-value of the conditional false discovery rate (condFDR) analysis between trait1 and trait 2. *P*1: Association *P* value for the GERD dataset. BETA1: Effect size estimate for the GERD dataset. *P*2: Association P value for the psychiatric disorder dataset. BETA2: Effect size estimate for the psychiatric disorder dataset. Gene.RefGene: Genes annotated by the RefGene database (reference gene database) that are associated with the lead SNP. GeneDetail.refGene: Detailed gene annotation, including the relative position to the SNP. cytoBand: Cytogenetic band location of the lead SNP.

**FIGURE 2:**
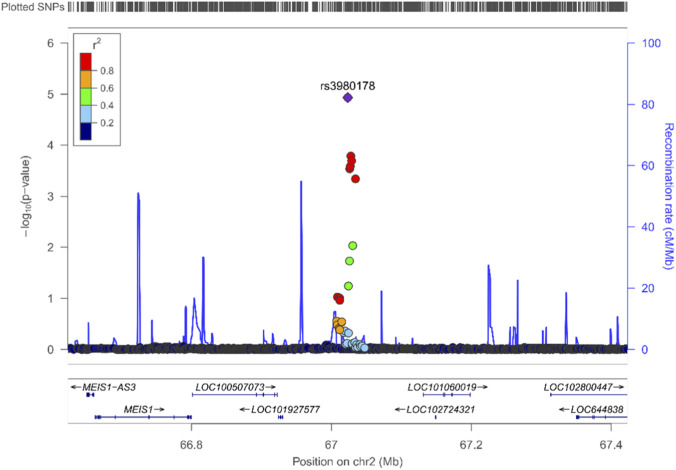
LocusZoom plot of GERD–MDD condFDR association signals at the rs3980178 locus. This plot shows the conditional false discovery rate (condFDR) association results for gastroesophageal reflux disease (GERD) conditioned on major depressive disorder (MDD). The lead SNP, rs3980178, which exceeds the condFDR significance threshold, is highlighted in purple. Surrounding SNPs are colored according to their linkage disequilibrium (LD; r^2^) with the lead variant, illustrating the regional LD structure. The blue line displays local recombination rates across the locus.

The GERD–MDD–associated variant rs3980178 lies in a regulatory region on chromosome 2 situated near *MEIS1*, a transcription factor involved in neurodevelopment, autonomic regulation, and central sensory processing. Although the variant is sub-genome-wide significant in GERD and MDD individually, its enrichment under the condFDR framework suggests pleiotropic regulatory influences shared across depressive and gastrointestinal phenotypes. The local association architecture (Figure 2) indicates a cluster of non-coding signals consistent with enhancer activity in neural tissues, supporting the hypothesis that *MEIS1*-linked regulatory mechanisms may modulate neural circuits relevant to affective function and visceral sensation—processes implicated in both GERD symptom generation and MDD risk.

**FIGURE 3:**
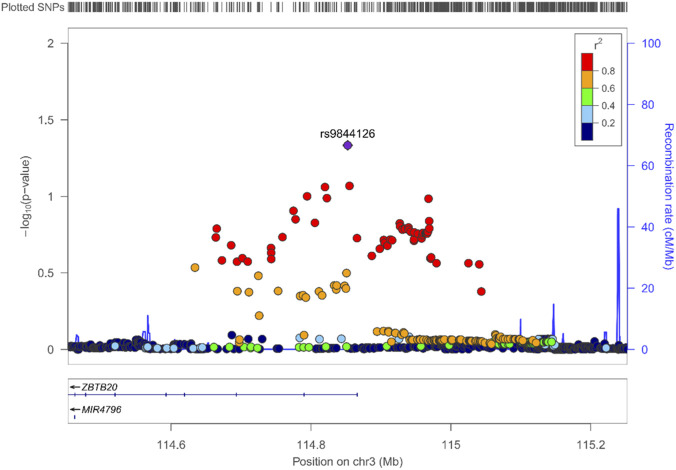
LocusZoom plot of GERD–SCZ condFDR association signals at the rs9844126 locus. This plot displays the conditional false discovery rate (condFDR) association results for gastroesophageal reflux disease (GERD) conditioned on schizophrenia (SCZ). The lead variant, rs9844126, which reaches condFDR significance, is highlighted in purple. SNPs across the region are colored by their linkage disequilibrium (LD; r^2^) with the lead SNP, illustrating the local LD structure. The blue curve represents the regional recombination rate.

The GERD–SCZ–associated variant rs9844126 is located on chromosome 3 within a region harboring *ZBTB20*, a transcriptional regulator essential for forebrain development and hippocampal maturation. GTEx data indicate that rs9844126 acts as an eQTL for *ZBTB20* in whole blood, suggesting that the variant may influence gene expression through systemic or immune-mediated pathways, while *ZBTB20*’s known roles in cortical–limbic circuit formation provide a mechanistic link to psychiatric vulnerability. The conditional enrichment observed between GERD and SCZ at this locus (Figure 3) raises the possibility that regulatory variation affecting *ZBTB20* contributes to shared biological processes such as stress responsivity, visceral–cortical signaling, or neuroimmune integration—domains relevant to both gastrointestinal function and schizophrenia liability.

## 3 Discussion

In this study, we investigated the shared genetic architecture between GERD and major psychiatric disorders specifically within East Asian populations, integrating ancestry-matched GWAS, cross-trait heritability analyses, and condFDR methods. Although single-trait GERD GWAS in East Asians lacked genome-wide significant findings—largely reflecting modest sample sizes relative to European consortia—our cross-phenotype analytic framework revealed previously unrecognized susceptibility loci by leveraging genetic enrichment with psychiatric traits. These results demonstrate that substantial biological insight can be gained even from underpowered datasets when multivariate genetic information is appropriately incorporated.

Our LDSC analyses highlight meaningful polygenic covariance between GERD and both MDD and SCZ in East Asians. This observation is consistent with extensive epidemiological evidence documenting elevated rates of depressive and psychotic symptoms among individuals with reflux-related disorders. Importantly, the presence of positive genetic correlations suggests that shared heritable liabilities—rather than environmental confounding alone—contribute to these comorbidity patterns. The absence of detectable genome-wide overlap with BIP underscores the specificity of these links and supports the notion that GERD shares genetic substrates selectively with certain psychiatric dimensions, particularly those related to mood regulation and cognitive–perceptual processing.

By applying condFDR, we identified two loci—near *MEIS1* and *ZBTB20*—that would not have been captured by single-trait GERD analyses. Both genes have established roles in neurodevelopment and higher-order brain functions, pointing to central neural pathways as potential mediators of GERD liability. *MEIS1* participates in autonomic network development and sensory integration, offering a plausible biological mechanism through which variation at this locus could influence esophageal sensation, pain perception, or reflexive motor control. Similarly, *ZBTB20* regulates cortical and hippocampal maturation, with downstream effects on stress responsivity, visceral perception, and neuroimmune signaling. The observation that rs9844126 is an eQTL for *ZBTB20* further supports the interpretation that regulatory variation affecting this transcription factor may shape both psychiatric vulnerability and GERD risk via shared neural or systemic pathways.

More broadly, these findings reinforce emerging evidence that gastrointestinal disorders with prominent sensory or pain components exhibit genetic and biological convergence with psychiatric traits. Such convergence likely reflects the intertwined nature of the neural circuits governing affect, interoception, autonomic function, and gastrointestinal physiology. By conducting these analyses in East Asian populations, our study also underscores the value of ancestry-specific genetic investigation. The loci identified here may represent risk variants that are more detectable—or perhaps even unique—in the East Asian genomic landscape due to population-specific allele frequencies and linkage disequilibrium patterns.

Several limitations warrant consideration. First, the available East Asian GERD GWAS datasets remain modest in sample size, which constrains statistical power for locus discovery and fine-mapping and likely contributes to the absence of genome-wide significant loci in the primary GERD GWAS meta-analysis. As a result, loci highlighted through downstream cross-trait analyses using condFDR should be interpreted as exploratory and hypothesis-generating, rather than definitive GERD risk loci, and will require confirmation in larger and independent GERD GWAS datasets. In addition, GERD is a clinically heterogeneous phenotype, and the contributing GWAS primarily relied on clinical diagnoses or standardized phenotype definitions within individual cohorts. Such heterogeneity may capture overlapping but not identical clinical presentations and is therefore expected to attenuate effect sizes and reduce sensitivity, rather than introduce spurious associations. Consequently, the shared genetic signals observed in this study are likely to reflect core biological components common across GERD definitions. Psychiatric traits, while substantially powered in some cases, also exhibit heterogeneity in ancestry composition and phenotypic ascertainment, which may influence the sensitivity of cross-trait analyses. Moreover, the cross-trait analyses in this study were limited to major depressive disorder, schizophrenia, and bipolar disorder, reflecting the availability of well-powered GWAS summary statistics in East Asian populations. Other relevant psychiatric and stress-related phenotypes, such as anxiety-related traits or ADHD, could not be examined and should be explored in future studies as suitable datasets become available. Finally, although genes such as MEIS1 and ZBTB20 were highlighted through integrative genetic analyses, the mechanistic links inferred here remain speculative and require experimental validation in relevant neural and peripheral tissues. Independent replication and functional validation using complementary datasets were not feasible given current data availability. Future studies incorporating larger multi-ancestry meta-analyses, refined phenotyping, and cell-type–specific functional genomics—particularly within brain–gut axis models—will be essential to further delineate causal pathways.

In summary, our study demonstrates that cross-trait genetic enrichment can substantially enhance locus discovery for GERD in East Asian populations and reveals shared neurobiological architecture with major depressive disorder and schizophrenia. These results provide a foundation for refining mechanistic models of GERD that incorporate central neural regulation and highlight the importance of considering psychiatric dimensions in understanding the genetic basis of gastrointestinal disorders.

## 4 Methods

### 4.1 GWAS data

Summary statistics for gastroesophageal reflux disease were obtained from two sources: the Taiwan Precision Medicine Initiative (47,138 cases and 271,934 controls) (Yang HC. et al., 2025) and a cross-population atlas of genetic associations (948 cases and 177,516 controls) (Sakaue et al., 2021). Psychiatric disorder datasets included Major Depressive Disorder (88,316 cases and 902,757 controls) (Meng et al., 2024), Schizophrenia (22,778 cases and 35,362 controls) (Lam et al., 2019), and Bipolar Disorder (158,036 cases and 2,800,000 controls) (O’Connell et al., 2025), sourced from published GWAS summary statistics repositories. Comprehensive details pertaining to each individual dataset are provided in Supplementary Table S1.

### 4.2 Estimation of genetic correlation

Genetic correlations (r_g_) between GERD and various psychiatric disorders were estimated using linkage disequilibrium score regression (LDSC) applied to GWAS summary statistics. Following standard protocols, the analysis was restricted to single-nucleotide polymorphisms (SNPs) included in the HapMap3 panel. Within the same LDSC framework, heritability estimates on the observed scale (h^2^) were simultaneously calculated for each trait. Importantly, LDSC explicitly models an intercept term, which allows the method to account for potential sample overlap and other sources of confounding between GWAS summary statistics, thereby providing unbiased estimates of genetic correlation even in the presence of modest overlap. The required linkage disequilibrium (LD) reference was obtained from the East Asian subset of the 1,000 Genomes Project, with specific extraction of HapMap3 variants (Bulik-Sullivan et al., 2015).

### 4.3 condFDR analysis

To augment the identification of GERD-associated loci and pinpoint variants jointly enriched in GERD and psychiatric phenotypes, we employed the conditional false discovery rate (condFDR) framework (Andreassen et al., 2014). This empirical Bayesian approach models cross-trait enrichment by integrating association signals from two GWAS datasets. It recalibrates test statistics for SNPs of the primary trait (GERD) based on their linkage to a secondary trait (e.g., MDD), effectively reprioritizing genetic markers and boosting detection power for true associations—even for variants below genome-wide significance. Importantly, condFDR operates entirely on GWAS summary statistics and leverages genome-wide enrichment patterns rather than individual-level data, rendering the approach relatively robust to modest sample overlap between studies. Conditional analyses were performed separately for each psychiatric phenotype, and results were interpreted in a trait-specific manner rather than as a pooled multi-trait test. To ensure reliable enrichment modeling, SNPs with secondary-trait P-values ≥ 0.05 were omitted from condFDR re-ranking. This filter retains variants showing at least modest association with the secondary trait, enhancing the stability of conditional FDR estimates and conforming to prior implementations. For the retained SNPs, condFDR was computed following standard protocol, and variants with condFDR < 0.05 were deemed conditionally significant, in line with conventional practice. In accordance with recommendations for Bayesian FDR modeling, regions of high linkage disequilibrium—specifically the extended MHC region (chr6: 25–34 Mb) and chromosome 8p23.1 (chr8: 7.2–12.5 Mb)—were excluded prior to model fitting.

## Data Availability

The original contributions presented in the study are included in the article/Supplementary Material, further inquiries can be directed to the corresponding authors.

## References

[B1] Anurag V. Jennifer H. Alex R. Peter M. Sarah L. David J. (2024). Diversity and scale: genetic architecture of 2068 traits in the va million veteran program. Sci. 10.1126/science.adj1182 PMC12857194 39024449

[B2] An J. Gharahkhani P. Law M. H. Ong J. S. Han X. Olsen C. M. (2019). Gastroesophageal reflux gwas identifies risk loci that also associate with subsequent severe esophageal diseases. Nat. Communications 10, 4219. 10.1038/s41467-019-11968-2 31527586 PMC6746768

[B3] Andreassen O. A. Thompson W. K. Dale A. M. (2014). Boosting the power of schizophrenia genetics by leveraging new statistical tools. Schizophr. Bulletin 40, 13–17. 10.1093/schbul/sbt168 24319118 PMC3885310

[B4] Azer S. A. Goosenberg E. (2025). “Gastroesophageal reflux disease (gerd),” in StatPearls. 32119349

[B5] Bulik-Sullivan B. K. Loh P. R. Finucane H. K. Ripke S. Yang J. Patterson N. (2015). LD score regression distinguishes confounding from polygenicity in genome-wide association studies. Nat. Genetics 47, 291–295. 10.1038/ng.3211 25642630 PMC4495769

[B6] Ding H. Jiang Y. Sun Q. Song Y. Dong S. Xu Q. (2025). Integrating genetics and transcriptomics to characterize shared mechanisms in digestive diseases and psychiatric disorders. Commun. Biology 8, 47. 10.1038/s42003-025-07481-6 39809838 PMC11733146

[B7] Lam M. Chen C. Y. Li Z. Martin A. R. Bryois J. Ma X. (2019). Comparative genetic architectures of schizophrenia in east asian and european populations. Nat. Genetics 51, 1670–1678. 10.1038/s41588-019-0512-x 31740837 PMC6885121

[B8] Meng X. Navoly G. Giannakopoulou O. Levey D. F. Koller D. Pathak G. A. (2024). Multi-ancestry genome-wide association study of major depression aids locus discovery, fine mapping, gene prioritization and causal inference. Nat. Genetics 56, 222–233. 10.1038/s41588-023-01596-4 38177345 PMC10864182

[B9] O’Connell K. S. Koromina M. van der Veen T. Boltz T. David F. S. Yang J. M. K. (2025). Genomics yields biological and phenotypic insights into bipolar disorder. Nature 639, 968–975. 10.1038/s41586-024-08468-9 39843750 PMC12163093

[B10] Sakaue S. Kanai M. Tanigawa Y. Karjalainen J. Kurki M. Koshiba S. (2021). A cross-population atlas of genetic associations for 220 human phenotypes. Nat. Genetics 53, 1415–1424. 10.1038/s41588-021-00931-x 34594039 PMC12208603

[B11] Smeland O. B. Frei O. Shadrin A. O'Connell K. Fan C. C. Bahrami S. (2019). Discovery of shared genomic loci using the conditional false discovery rate approach. Hum. Genet. 139, 85–94. 10.1007/s00439-019-02060-2 31520123

[B12] Song Y. Li L. Jiang Y. Peng B. Jiang H. Chao Z. (2025). Multitrait genetic analysis identifies novel pleiotropic loci for depression and schizophrenia in east asians. Schizophr. Bulletin 51, 684–695. 10.1093/schbul/sbae145 39190819 PMC12061663

[B13] Wang D. Liu S. Wu Q. Jiang Y. Zhang C. Ye W. (2025). Identification of shared genetic loci for asthma, allergic rhinitis, and pollinosis in east asians. Sci. Reports 15, 6068. 10.1038/s41598-025-90443-z 39972113 PMC11840148

[B14] Yang L. Peng B. Wu Y. Wang X. Zhang Q. Chen J. (2025a). Uncovering pleiotropic loci in allergic rhinitis and leukocyte traits through multi-trait gwas. Sci. Reports 15, 23057. 10.1038/s41598-025-07100-8 40593228 PMC12218862

[B15] Yang H. C. Kwok P. Y. Li L. H. Liu Y. M. Jong Y. J. Lee K. Y. (2025b). The Taiwan precision medicine initiative provides a cohort for large-scale studies. Nature 648, 117–127. 10.1038/s41586-025-09680-x 41092961 PMC12675286

[B16] Zhi L. Zheng Q. Jiang Y. Yu L. Li L. Song Y. (2025). Multi-trait genetic analysis of asthma and eosinophils uncovers pleiotropic loci in east asians. Nat. Commun. 16, 5081. 10.1038/s41467-025-60405-0 40450010 PMC12126482

